# 芦曲泊帕治疗环孢素A难治性输血依赖型非重型再生障碍性贫血的疗效与安全性

**DOI:** 10.3760/cma.j.cn121090-20250225-00094

**Published:** 2025-10

**Authors:** 永鑫 周, 扬阳 魏, 紫薇 刘, 辰 杨, 苗 陈, 冰 韩

**Affiliations:** 1 中国医学科学院北京协和医院血液科，北京 100730 Department of Hematology, Chinese Academy of Medical Science and Peking Union Medical College Hospital, Beijing 100730, China; 2 中国医学科学院北京协和医学院，北京 100730 School of Clinical Medicine, Chinese Academy of Medical Sciences and Peking Union Medical College, Beijing 100730, China

## Abstract

回顾性分析12例接受芦曲泊帕单药治疗的环孢素A（CsA）难治性输血依赖型非重型再生障碍性贫血（TD-NSAA）患者，全部患者均伴有多种慢性基础疾病或药物相关风险，或对其他血小板受体激动剂（TPO-RA）无效或不耐受。芦曲泊帕中位治疗4（3～11）个月，中位随访8（6～11）个月。第3个月、第6个月及随访期末的治疗反应（OR）率分别为50.0％、58.3％和50.0％，OR中位起效时间2（1～4）个月；完全治疗反应（CR）率分别为8.3％、16.7％和16.7％，CR中位起效时间4（2～5）个月。不良反应均为Ⅰ级，发生率为25.0％。随访期间，1例停药后未能维持OR，复发率14.3％；未见克隆演变或死亡。提示芦曲泊帕对CsA难治性TD-NSAA患者有效且耐受性良好。

## Abstract (en)

This study retrospectively analyzed 12 patients with transfusion-dependent, non-severe aplastic anemia（TD-NSAA）refractory to cyclosporine A（CsA）, who were treated with lusutrombopag monotherapy. These patients either had a variety of chronic comorbidities or medication-related risks, or they were unresponsive to or intolerant of other thrombopoietin receptor agonists（TPO-RA）. The median treatment duration with lusutrombopag was 4 months（range: 3–11 months）, while the median follow-up period was 8 months（range: 6–11 months）. The overall response（OR）rates at months 3, 6, and the end of follow-up were 50.0％, 58.3％, and 50.0％, respectively, with a median time to OR of 2 months（range: 1–4 months）. Complete response（CR）rates were 8.3％, 16.7％, and 16.7％ at the same time points, with a median time to CR of 4 months（range: 2–5 months）. Adverse events were all Grade 1, with an incidence rate of 25.0％. During follow-up, one patient experienced a loss of OR after discontinuing treatment, with a relapse rate of 14.3％; no clonal evolution or mortality was observed. These findings suggest that lusutrombopag is both effective and well-tolerated in CsA-refractory TD-NSAA patients and represents a promising therapeutic option for those with poor treatment tolerability.

再生障碍性贫血（AA）是一种以骨髓造血功能衰竭和外周全血细胞减少为特征的疾病[1]。输血依赖型非重型AA（TD-NSAA）约占AA住院患者的三分之二，部分易进展为重型AA（SAA）[2]。对无HLA相合同胞供者和年龄>40岁的TD-NSAA患者，首选免疫抑制剂（IST）［抗胸腺/淋巴细胞球蛋白（ATG/ALG）+环孢素A（CsA）］联合血小板生成素受体激动剂（TPO-RA）治疗[3]。但因ATG/ALG需住院治疗且存在输液反应和肝功能损伤风险，实际应用中IST以CsA单药为主。目前中国获批AA适应证的TPO-RA仅有艾曲泊帕和海曲泊帕，但两者均有潜在的肝毒性[4]–[5]，在肝肾功能异常或顾虑药物相互作用的患者中使用受限。此外，还有部分患者对足量艾曲泊帕或海曲泊帕治疗无效，长期输血依赖严重影响生活质量。

芦曲泊帕作为新一代口服TPO-RA[6]，安全性较高[7]，在血小板减少疾病中疗效显著[8]–[9]。尽管尚未获批AA适应证，芦曲泊帕可能为肝肾功能异常或其他TPO-RA无效患者提供替代选择。目前尚缺乏芦曲泊帕治疗CsA难治性TD-NSAA患者的研究。本研究旨在评估芦曲泊帕在该类患者中的疗效与安全性。

## 病例与方法

1. 病例：本研究为单中心、回顾性、病例系列研究，分析2024年1月至6月在北京协和医院连续接受芦曲泊帕单药治疗≥3个月的12例CsA难治性TD-NSAA成年患者临床资料。TD-NSAA诊断及成分血输注指征均参照《再生障碍性贫血诊断与治疗中国指南（2022年版）》[3]。CsA难治性定义为经足量CsA治疗≥6个月无效[10]。入选患者前序治疗已停药≥3个月，且至少符合下列条件之一：①合并多种慢性基础疾病；②TPO-RA合用基础疾病用药存在风险；③对其他TPO-RA无效（足量使用≥3个月）或不耐受。本研究获北京协和医院伦理委员会批准（批件号：K7874）。

2. 治疗方案：芦曲泊帕起始剂量6 mg/d，PLT恢复正常后可逐渐减量。ANC<0.5×10^9^/L时使用粒细胞集落刺激因子（G-CSF）5 µg·kg^−1^·d^−1^。感染时可合用敏感抗生素。未合用CsA、雄激素等药物。

3. 随访：随访截止日期为2025年1月，中位随访8（6～11）个月。通过查阅住院病历及门诊记录收集相关数据，必要时致电当地医师补充信息。主要随访指标包括血常规（含网织红细胞）、生化指标（肝、肾功能及贫血三项）等。随访时间点包括但不限于治疗开始后第1、2、3和6个月。

4. 疗效与安全性评估：疗效标准参照文献[3]。复发定义为达到且维持有治疗反应（OR）≥3个月后至少1种血系出现实质性下降，需重新调整芦曲泊帕剂量[11]。不良事件分级依据NCI-CTC5.0版。

5. 统计学处理：计量资料以中位数（范围）、计数资料以例数（构成比）进行描述。

## 结果

一、基线资料

12例CsA难治性TD-NSAA患者基线特征如表1所示。无一例患者伴PNH克隆；基础疾病包括糖尿病、慢性肾病、高血压、心脏病和乙型肝炎，需要治疗并存在合并用药风险。

**表1:** 12例环孢素A难治性输血依赖型非重型再生障碍性贫血患者的基线特征

例号	性别	年龄（岁）	血细胞减少	输血依赖	HGB（g/L）	PLT（×10⁹/L）	ANC（×10⁹/L）	ARC（×10⁹/L）	ALT（U/L）	TBIL（µmol/L）	肌酐（µmol/L）	血清铁蛋白（µg/L）	基础疾病
1	男	18	三系	红细胞、血小板	55	0.55	6	30.1	88	13.9	56	2723	无
2	女	43	三系	红细胞	37	0.58	24	23.6	117	12.4	81	3632	无
3	女	61	两系	血小板	96	3.06	9	74.7	197	17.2	67	259	糖尿病
4	男	62	两系	血小板	124	1.36	10	76.5	93	17.1	71	192	糖尿病
5	女	24	三系	血小板	86	0.75	5	46.6	81	15.3	58	202	无
6	男	51	两系	血小板	105	0.68	14	69.5	134	16.4	58	766	无
7	女	72	两系	血小板	105	1.18	9	75.1	45	15.6	58	867	心脏病
8	男	58	两系	血小板	88	1.68	11	64.9	144	14.1	104	493	糖尿病、乙型肝炎
9	女	58	两系	血小板	117	0.85	18	77.6	127	14.8	70	197	高血压
10	女	40	两系	血小板	78	1.79	12	55.2	131	12.4	59	553	无
11	男	63	三系	血小板	76	0.61	7	47.1	126	16.1	175	1391	慢性肾病
12	女	68	三系	血小板	81	0.55	14	45.5	90	13.9	65	199	糖尿病、慢性肾病

**注** ARC：网织红细胞绝对计数；ALT：丙氨酸转氨酶；TBIL：总胆红素

患者既往用药情况如图1所示，包括：CsA 12例（100.0％），中位治疗时间9（6～20）个月；他克莫司5例（41.7％），中位治疗时间7（2～28）个月；艾曲泊帕1例（8.3％），治疗时间24个月；海曲泊帕6例（50.0％），中位治疗时间6（3～9）个月；既往TPO-RA治疗时均未与CsA或雄激素合用。5例（41.7％）患者既往未经TPO-RA治疗。

**图1:**
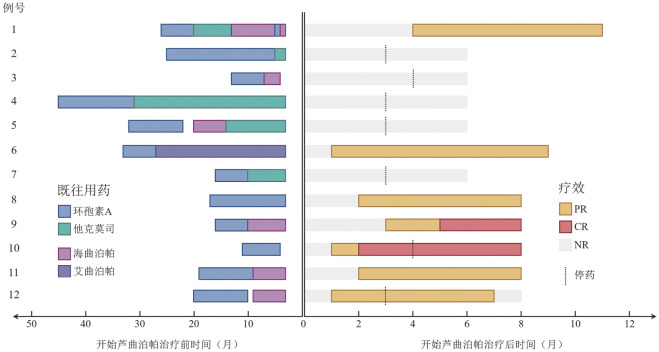
12例患者的既往用药及芦曲泊帕治疗反应泳道图 **注** CR：完全治疗反应；PR：部分治疗反应；NR：无治疗反应

二、疗效分析

芦曲泊帕中位治疗4（3～11）个月，中位剂量6（3～6）mg/d。治疗反应情况如图1所示。第3个月OR率（ORR）和完全治疗反应（CR）率分别为50.0％和8.3％。7例（58.3％）患者治疗<6个月：5例因疗效欠佳，1例因达到OR，1例因达到CR。第6个月ORR和CR率分别为58.3％和16.7％。治疗≥6个月的5例（41.7％）患者均达到OR。随访期末，停药的OR患者丧失疗效，ORR和CR率分别为50.0％和16.7％。OR和CR中位起效时间分别为2（1～4）个月和4（2～5）个月。

血系应答情况如图2所示（包括已停药患者的随访数据）。芦曲泊帕治疗开始后第3个月，33.3％一系应答，16.7％两系应答，无三系应答；第6个月，33.3％一系应答，25.0％两系应答，无三系应答；随访期末，25.0％一系应答，16.7％两系应答，8.3％三系应答。血红蛋白、中性粒细胞、血小板应答中位起效时间分别为2（1～5）个月、5（2～9）个月和2（1～4）个月。

**图2:**
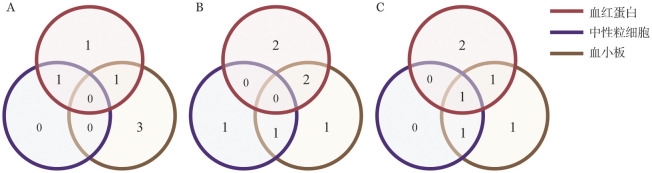
芦曲泊帕治疗血系应答情况（维恩图展示了一系、两系和三系应答的患者数 **A** 治疗后第3个月；**B** 治疗后第6个月；**C** 随访期末

进一步比较既往接受其他TPO-RA治疗无效（7例）与未经TPO-RA治疗（5例）的患者。在第3个月、第6个月及随访期末，两组患者ORR分别为57.1％对40.0％、71.4％对40.0％及57.1％对40.0％；CR率分别为0对20.0％、14.3％对20.0％及14.3％对20.0％。两组患者OR和CR中位起效时间分别为2个月对2个月，以及5个月对2个月。

三、安全性分析

随访期内，共有3例（25.0％）患者出现Ⅰ级不良反应，包括头痛（16.7％）、食欲下降（8.3％）和白细胞减少（8.3％）；其中1例患者同时出现头痛和食欲下降。未见患者因不良事件减量、停药或死亡。

四、复发、克隆演变和转归

随访期内，复发率为14.3％（1例患者因达到OR，在用药3个月后停药，但停药后第5个月未能维持OR）；未见克隆演变或死亡。

## 讨论

芦曲泊帕尚未获批AA适应证。本研究选择芦曲泊帕治疗的原因包括：①基础疾病导致潜在脏器功能代偿性差，预计使用艾曲泊帕和海曲泊帕耐受性差；②基础疾病用药与其他TPO-RA存在合并用药风险；③患者既往使用其他TPO-RA疗效或耐受性不佳。

本研究首次评估了芦曲泊帕治疗CsA难治性TD-NSAA的疗效与安全性。第3个月、第6个月和随访期末的ORR分别为50.0％、58.3％和50.0％，与艾曲泊帕在类似患者中的疗效相似（艾曲泊帕单药治疗难治性TD-NSAA第3个月和第6个月的ORR分别为51.2％和58.5％）[12]。OR中位起效时间为2（1～4）个月，与艾曲泊帕治疗起效时间相似［艾曲泊帕治疗TD-NSAA的OR中位起效时间为3（1～6）个月］[12]。

与既往研究不同，本研究包含一部分既往接受其他TPO-RA治疗无效的患者。即便如此，芦曲泊帕仍展现出良好的疗效。至少提示，艾曲泊帕或海曲泊帕治疗无效的部分患者，更换为芦曲泊帕可能还有一定疗效。

在结合胞内区的口服TPO-RA中，芦曲泊帕的药物相互作用最小[13]–[14]，且不良反应较小[9]，适合合并慢性基础疾病、老年、肝肾功能代偿能力弱，或合并用药多的患者。本研究中，芦曲泊帕的不良反应均为Ⅰ级，发生率为25.0％；观察到的不良事件包括头痛、食欲下降和白细胞减少，与既往芦曲泊帕治疗其他血小板减少的报道类似[15]–[16]；未见治疗相关减量、停药或死亡。相比之下，艾曲泊帕治疗相关不良反应发生率为39.0％，12.2％出现Ⅲ级不良反应，表现为严重的恶心和心动过速[12]。此外，艾曲泊帕还带有FDA肝毒性黑框警告。以上数据表明，芦曲泊帕在耐受性和安全性上具有一定优势。

文献报道，28（12～48）个月随访期内，艾曲泊帕治疗的复发率为21.2％，克隆演变率为10.9％[17]。本研究复发率为14.3％，未见克隆演变。上述结果可能与随访时间较短有关，未来需开展更长随访时间及更大样本量的研究，以进一步明确芦曲泊帕治疗的复发与克隆演变风险。

本研究为回顾性研究，样本量和随访时间有限，且难以分析影响应答的可能因素；但初步验证了芦曲泊帕在CsA难治性TD-NSAA患者中的疗效与安全性。结果表明，对伴有多种基础疾病、存在合并用药风险、或既往对其他TPO-RA无效或不耐受的患者，芦曲泊帕可能是一种有效且安全的治疗选择。
